# Twists to Classical Conditioning of Adult *Drosophila*

**DOI:** 10.1101/pdb.top108567

**Published:** 2025-08-01

**Authors:** Zeynep Okray, Scott Waddell

**Affiliations:** Centre for Neural Circuits and Behaviour, University of Oxford, Oxford OX1 3TA, UK

## Abstract

Memory has been extensively studied in *Drosophila* since the early 1970s. Straightforward aversive and appetitive conditioning paradigms train populations of flies to associate the pairing of one of two odors with either punishment or reward. After training, the flies show either preferential avoidance or approach behavior, to the appropriate odor, when given a choice between the two odors in a simple T-maze apparatus. These basic experimental approaches have proven useful in understanding the genetic, molecular, cellular, and neuronal network bases of various valence-specific memories in the fly brain. In addition, numerous modifications to these assays have permitted the study of a broad range of memory-related phenomena. Labile short-term avoidance and approach memories can be readily distinguished from more stable “consolidated” long-term memory equivalents. Prior or sub-sequent experience of the training cues, and manipulations of the flies’ condition, have revealed how parallel competing memories and incompatible states can temporarily interfere with memory retrieval, providing insight into mechanisms of forgetting. Recent studies have also modified the training and testing apparatus to allow simultaneous presentation of odors and colors, providing insight into mechanisms of multisensory learning.

## Introduction

Investigation of *Drosophila* memory was first reported in the early 1970s by William G. “Chip” Quinn and William A. Harris under the tutelage of Seymour Benzer (Quinn et al. 1974). These studies were initiated with the goal of identifying genes that encode critical elements of the learning machinery. Over the years, approaches and goals have shifted. Thousands of individual fly lines are now available that allow investigators to precisely control gene expression in many (most, even) individual cell types in the fly brain, especially within memory-relevant networks of the mushroom bodies (Pfeiffer et al. 2010; Gohl et al. 2011; Jenett et al. 2012; Aso et al. 2014; Tirian and Dickson 2017; Luan et al. 2020). Using these “neuron-specific” lines with a variety of powerful effector transgenes permits one to label and visualize (Lee and Luo 1999; Sutcliffe et al. 2017), manipulate the expression of specific genes within (Dietzl et al. 2007; Hu et al. 2021), record from (Chamberland et al. 2017; Jing et al. 2020; Sun et al. 2020; Kannan et al. 2022; Abdelfattah et al. 2023; Zhang et al. 2023), and control the activity of (Kitamoto 2001; Lima and Miesenbock 2005; Hamada et al. 2008; Klapoetke et al. 2014; Mohamed et al. 2017; Ott et al. 2024) the neurons of interest. With these tools in hand, memory research in the fly now extends past traditional neuroscience boundaries to include molecular, cellular, systems, and behavioral studies (Cognigni et al. 2018; Modi et al. 2020). Our associated protocol describes the manufacture and use of a T-maze apparatus to test aversive and appetitive olfactory and multisensory learning and memory in *Drosophila* (see Protocol: Classical Conditioning of Adult *Drosophila* [Okray et al. 2024]).

## Aversive Conditioning

The aversive conditioning assay that is used in many laboratories was initially devised by Tim Tully in Chip Quinn’s laboratory (Tully and Quinn 1985). Approximately 100 flies are trained for 1 min to associate one of two odors with 12 electric shocks. When later given a choice between two T-maze arms suffused with either the previously shock-paired odor (CS^+^) or the other odor (CS^−^), the flies preferentially avoid the CS^+^. A single 1-min training session forms CS^+^ avoidance memory that is barely detectable when performance is measured this way 24 h afterwards.

It is generally appreciated that the perdurance of measurable memory depends on the training protocol. A seminal study showed that five to 10 repetitions of the 1-min aversive training session can extend the time in which “aversive” memory can be measured to >1 wk, and this was most apparent if the sessions are “spaced” with intervening rest intervals, rather than one after the other—”massed” (Tully et al. 1994). This work introduced the concept of two coexisting types of genetically and pharmacologically separable long-term aversive memory: anesthesia-resistant memory (ARM) and protein synthesis-dependent long-term memory (LTM). A later paper argued that ARM and LTM were mutually exclusive (Tully et al. 1994).

### Varying Test Odor Choice and Context

Recently, instead of giving flies a choice of CS^+^ versus CS^−^ after testing, flies were given a choice of CS^+^ versus a novel odor (one they had never experienced in training), or CS^−^ versus a novel odor. This work found that repetitive CS^+^/CS^−^ training trials actually allow flies to learn more information than just an odor–shock association (Jacob and Waddell 2020). Most evidently, the performance gain from spaced aversive training (CS^+^/CS^−^ trials) results from flies learning that the previously shock-paired CS^+^ should be avoided but also that the explicitly non-shock-paired CS^−^ odor is “safe.” Perhaps surprisingly, the CS^−^ safety memory appears to account for the most persistent odor LTM following spaced aversive training, whereas ARM represents the aversive memory for the CS^+^.

A similarly surprising discovery was made when investigators kept the “context” of the trained odors consistent between training and testing. In regular aversive training and testing, flies are trained in a “shock” tube whose walls are an electrifiable copper grid and are tested for odor preference in a different context: The two testing tubes are clear plastic. However, when Zhao et al. (2019) instead tested trained flies for their odor preference using two nonelectrified shock tubes, they observed that even a single 2-min aversive training session formed memory measurable for up to 2 wk! Therefore, a single training session forms long-lasting memories, but their retrieval is context-dependent. This finding suggests that repetitive training also permits flies to form odor valence memories that are “free” of the context constraints that are a property of memory following a single training trial.

Another recent study added additional evidence that the single training session forms long-lasting aversive memories. Up to 8 d after a single aversive training session, “forgotten” aversive memories could be reinstated by mild retraining, which is in itself insufficient to form measurable memory (Wang et al. 2023). This memory “savings” type of experiment (Ebbinghaus 1885) suggests that the new suboptimal learning adds to a memory that was there but could not be accessed.

## Appetitive Conditioning

The standard appetitive conditioning assay was initially devised by Bruce Tempel in Chip Quinn’s laboratory (Tempel et al. 1983). Here, populations of food-deprived flies are trained for 2 min to associate one of two odors with sucrose reward (Krashes and Waddell 2008; Colomb et al. 2009).

When later tested in the T-maze for their preference between the two odors used in training, the flies preferentially approach the previously sugar-paired odor. A single 2-min training session with sucrose reward forms protein synthesis-dependent LTM that remains measurable for days after.

An advantage of the sucrose reward paradigm is that sugar is a physiologically relevant reinforcer. It is also simple to change the odor-paired sugar from sucrose to those with different properties, such as other naturally occurring sugars that are more or less sweet and/or nutritious, or nonmetabolizable L-sugars, synthetic sweeteners, etc., to investigate different forms of memory (Burke and Waddell 2011; Burke et al. 2012; Huetteroth et al. 2015; McGinnis et al. 2016).

Complexities of the sucrose reward assay arise when measuring extended memory. Long-term food deprivation is obviously lethal. Therefore, one can only test memory beyond 24 h in the few flies that remain alive (if they were starved for 16 h before and 24 h after training), or feed the flies after training (Krashes and Waddell 2008). Although it might seem trivial to include an after-training feed, there are two critical considerations if choosing to do so. First, feeding to satiety posttraining suppresses memory performance. This is, however, reversible by restarving the flies prior to testing (Krashes and Waddell 2008; Krashes et al. 2009). The clear state dependence of sugar reward memory expression means that it is critical to distinguish between partially satiated flies and those with poor memory. The second issue to consider if feeding flies after sugar reward training is that neural processing of the resulting memory can be different in hungry and satiated conditions (Chouhan et al. 2021).

Flies can also be appetitively conditioned when thirsty by pairing odor and water reward (Lin et al. 2014; Lee et al. 2020). Expression of water-reinforced appetitive memories is similarly deprivation state-dependent but is specifically promoted by thirst rather than hunger. In fact, flies that are trained when both hungry and thirsty by pairing odor A with water and odor B with sugar will subsequently selectively seek odor A when thirsty and odor B when hungry (Senapati et al. 2019). This impressive level of state-dependent control of memory expression arises from neural circuit integration of internal state information with that for the appropriate memory (Krashes et al. 2009; Albin et al. 2015; Perisse et al. 2016; Senapati et al. 2019; Meschi et al. 2024).

## Further Twists On Learning and Memory Paradigms

Recent studies have described protocols to train flies to associate a specific odor and color combination with sucrose reward or with electric shock punishment (Thiagarajan et al. 2022; Okray et al. 2023), allowing study of multisensory learning. Interestingly, multisensory training enhances memory performance for the combined and individual odor and color sensory cues.

Aversive and appetitive paradigms in the fly have also been used to investigate neural mechanisms of other classical learning phenomena. For example, pre-exposing flies to an odor, before appetitive training with that odor and sucrose, can produce a temporary and context-dependent latent inhibition (Jacob et al. 2021). The flies learn the new odor–sugar association, but the retrieval of that memory is temporarily suppressed by competition with the prior odor pre-exposure memory, so no memory performance can be measured. Similarly, re-exposure to the previously reinforced CS^+^ odor *after* training triggers memory extinction, where the flies form a parallel memory of opposite valence that competes with the original memory and so temporarily nullifies memory performance (Felsenberg et al. 2017,^2018^; Yang et al. 2023). In contrast, re-exposure to the CS^−^ after training can trigger reconsolidation of the original memory, where it temporarily returns to a “nonconsolidated” form that can be disrupted with anesthesia (Felsenberg et al. 2017). In addition, presenting flies with a distracting air puff, shocks, or bright light before testing can temporarily inhibit memory expression, or induce “transient forgetting” (Sabandal et al. 2021).

Last, flies can also be trained by pairing an odor with direct activation of sensory or reinforcing neurons, rather than with sugar or shock/bitter (Schroll et al. 2006; Claridge-Chang et al. 2009; Aso et al. 2010; Burke et al. 2012; Liu et al. 2012; Das et al. 2014; Huetteroth et al. 2015; Jovanoski et al. 2023). Training in these cases involves presenting one of the two odors with a hot air stream (thermogenetics) or the appropriate wavelength of light (optogenetics) to trigger the genetically encoded ion channels, and thereby neuronal activation.

The approaches outlined above have been instrumental to progress in the field, having been successfully used to discover genes and neural circuit principles directing learning and memory. For further information, we refer readers to reviews by Waddell and Quinn (2001), Keene and Waddell (2007), Cognigni et al. (2018), Modi et al. (2020), and Davis (2023).
